# A Human IRE1 Inhibitor Blocks the Unfolded Protein Response in the Pathogenic Fungus Aspergillus fumigatus and Suggests Noncanonical Functions within the Pathway

**DOI:** 10.1128/mSphere.00879-20

**Published:** 2020-10-21

**Authors:** José P. Guirao-Abad, Martin Weichert, Aaron Albee, Katie Deck, David S. Askew

**Affiliations:** a Department of Pathology & Laboratory Medicine, University of Cincinnati College of Medicine, Cincinnati, Ohio, USA; University of Georgia

**Keywords:** *A. fumigatus*, UPR, 4μ8C, ER stress, IRE1, IreA, XBP1, Hac1, HacA, secretion, RNase, STF-083010, Ire1, Xbp1

## Abstract

The unfolded protein response (UPR) is a signaling network that maintains homeostasis of the endoplasmic reticulum (ER). In the human-pathogenic fungus Aspergillus fumigatus, the UPR is initiated by activation of an endoribonuclease (RNase) domain in the ER transmembrane stress sensor IreA, which splices the downstream mRNA *hacA^u^* into its active form, *hacA^i^,* encoding the master transcriptional regulator of the pathway. Small-molecule inhibitors against IRE1, the human ortholog of IreA, have been developed for anticancer therapy, but their effects on the fungal UPR are unexplored. Here, we demonstrate that the IRE1 RNase inhibitor 4μ8C prevented A. fumigatus from increasing the levels of *hacA^i^* mRNA, thereby blocking induction of downstream UPR target gene expression. Treatment with 4μ8C had minimal effects on growth in minimal medium but severely impaired growth on a collagen substrate that requires high levels of hydrolytic enzyme secretion, mirroring the phenotype of other fungal UPR mutants. 4μ8C also increased sensitivity to carvacrol, a natural compound that disrupts ER integrity in fungi, and hygromycin B, which correlated with reduced expression of glycosylation-related genes. Interestingly, treatment with 4μ8C was unable to induce all of the phenotypes attributed to the loss of the canonical UPR in a Δ*hacA* mutant but showed remarkable similarity to the phenotype of an RNase-deficient IreA mutant that is also unable to generate the *hacA^i^* mRNA. These results establish proof of principle that pharmacological inhibition of the canonical UPR pathway is feasible in A. fumigatus and support a noncanonical role for the *hacA^u^* mRNA in ER stress response.

**IMPORTANCE** The unfolded protein response (UPR) is a signaling pathway that maintains endoplasmic reticulum (ER) homeostasis, with functions that overlap virulence mechanisms in the human-pathogenic mold Aspergillus fumigatus. The canonical pathway centers on HacA, its master transcriptional regulator. Translation of this protein requires the removal of an unconventional intron from the cytoplasmic mRNA of the *hacA* gene, which is achieved by an RNase domain located in the ER-transmembrane stress sensor IreA. Here, we show that targeting this RNase activity with a small-molecule inhibitor effectively blocked UPR activation, resulting in effects that mirror the consequences of genetic deletion of the RNase domain. However, these phenotypes were surprisingly narrow in scope relative to those associated with a complete deletion of the *hacA* gene. These findings expand the understanding of UPR signaling in this species by supporting the existence of noncanonical functions for the unspliced *hacA* mRNA in ER stress response.

## Abstract (precis)

The unfolded protein response (UPR) is a signaling pathway that maintains endoplasmic reticulum (ER) homeostasis, with functions that overlap virulence mechanisms in the human-pathogenic mold Aspergillus fumigatus. The canonical pathway centers on HacA, its master transcriptional regulator. Translation of this protein requires the removal of an unconventional intron from the cytoplasmic mRNA of the *hacA* gene, which is achieved by an RNase domain located in the ER-transmembrane stress sensor IreA. Here, we show that targeting this RNase activity with a small-molecule inhibitor effectively blocked UPR activation, resulting in effects that mirror the consequences of genetic deletion of the RNase domain. However, these phenotypes were surprisingly narrow in scope relative to those associated with a complete deletion of the *hacA* gene. These findings expand the understanding of UPR signaling in this species by supporting the existence of noncanonical functions for the unspliced *hacA* mRNA in ER stress response.

## INTRODUCTION

**FIG 1:**
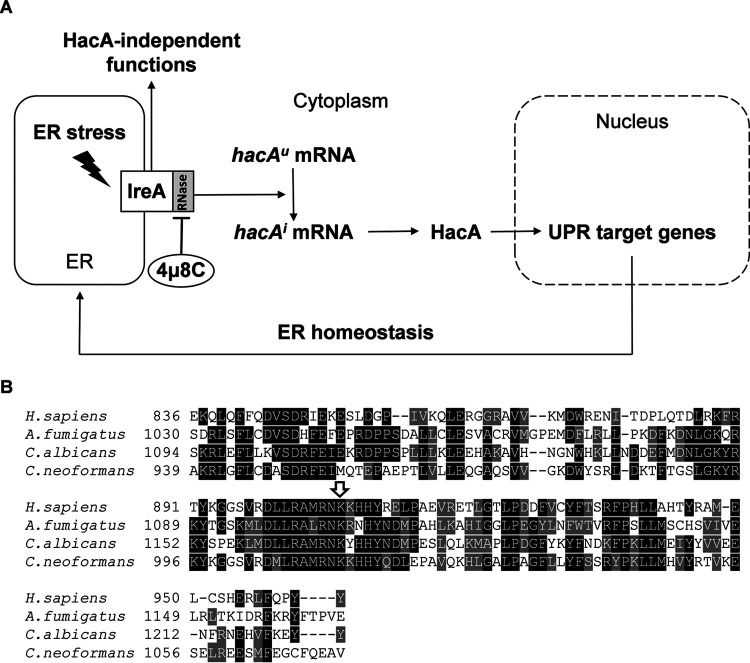
Schematic representation of the UPR pathway in A. fumigatus and alignment of the amino acid sequence of the RNase domain of human IRE1 with orthologs in fungi. (A) The UPR is activated by ER stress, which occurs when the demand for secretion exceeds the protein folding capacity of the ER or when the cell encounters stimuli that disrupt ER homeostasis. The resulting accumulation of unfolded proteins activates the cytosolic RNase domain of the ER transmembrane stress sensor IreA, which removes an intron from the uninduced mRNA *hacA^u^*, converting it into the induced mRNA *hacA^i^*, which is translated into the bZIP transcription factor HacA that coordinates a transcriptional program to augment ER folding capacity. The small-molecule inhibitor 4μ8C blocks the activity of the IreA RNase domain required for *hacA* mRNA processing. (B) Alignment of the RNase domain of human IRE1 with the corresponding region in A. fumigatus, C. albicans, and C. neoformans. The arrow indicates the conserved lysine residue targeted by 4μ8C. Black shading indicates identity to the column consensus and gray indicates similarity.

The unfolded protein response (UPR) is a eukaryotic signaling network that communicates information on the protein folding environment of the endoplasmic reticulum (ER) to the nucleus (1). In fungi, studies on the UPR have demonstrated that several species that infect plants, animals, or humans have uniquely adapted this stress response pathway to support the expression of pathogenicity traits, such as antifungal drug resistance, nutrient acquisition, host-temperature adaptation, cell wall or membrane homeostasis, and effector protein secretion (2–12). The pathway detects and responds to the accumulation of unfolded proteins, which is a situation that arises whenever the demand for increased output by the secretory pathway exceeds the protein folding capacity of ER-resident chaperones and folding enzymes (13). When unfolded proteins reach a critical threshold in the ER lumen, they activate an ER transmembrane protein known in humans as IRE1 (IreA in Aspergillus fumigatus [Fig. 1]). IRE1/IreA directs the most evolutionarily conserved branch of the UPR, and the basic paradigm of signaling is conserved across most fungal species, including A. fumigatus (14).

All species homologs of the IRE1 protein contain an ER luminal stress-sensing domain and a cytosolic effector region containing kinase and endoribonuclease (RNase) domains (15, 16). Interactions between the sensor and unfolded proteins trigger oligomerization in the ER membrane, resulting in *trans*-autophosphorylation and activation of the RNase domain. The RNase then removes an unconventional intron from a cytoplasmic mRNA that is known in A. fumigatus as *hacA^u^* (uninduced), creating a frameshift in the activated form of the mRNA, *hacA^i^* (induced), that is an obligatory step for the translation of the bZIP transcription factor HacA (1, 3, 5) (Fig. 1A). This master transcriptional regulator of the canonical UPR pathway helps to buffer physiological fluctuations in ER stress by orchestrating a transcriptional program that augments the folding of proteins trafficking through the secretory pathway (3, 13, 17).

Selective inhibitors of the kinase or RNase domain of human IRE1 have been developed as tools to experimentally dissect the contribution of the UPR to cell physiology and human disease, particularly with respect to cancer (18–24). Among these compounds, salicylaldehyde-based inhibitors have shown high efficacy by directly blocking the RNase domain (25). The compounds 4μ8C and STF-083010 are two well-described examples of this class (26–29), but their effects on a fungal pathogen are still unexplored. Here, we demonstrate that 4μ8C (30) is an inhibitor of the canonical UPR pathway in A. fumigatus, effectively blocking the accumulation of *hacA^i^* mRNA and downstream target gene induction. We found that treatment with 4μ8C failed to recapitulate the entire collection of phenotypes associated with a Δ*hacA* deletion mutant but was similar to phenotypes displayed by an IreA RNase domain mutant that is unable to process *hacA^u^* mRNA into *hacA^i^*. These data demonstrate the feasibility of pharmacological disruption of the canonical UPR pathway in a fungal pathogen and support emerging evidence that both the unspliced and spliced *hacA* mRNAs have functions in ER stress responses.

## RESULTS

### The human IRE1 RNase inhibitor 4μ8C blocks the canonical UPR of A. fumigatus.

We previously showed that the canonical UPR pathway of A. fumigatus involves a linear order of molecular events triggered by the activation of IreA in the presence of ER stress (3). Upon activation, the endoribonuclease domain of IreA splices an intron from the uninduced cytoplasmic mRNA *hacA^u^*, converting it to its induced form, *hacA^i^*, which is subsequently translated into the encoded transcription factor HacA (Fig. 1A). Subsequent studies revealed that deleting the *ireA* gene, or the ortholog in other fungal species (5, 31), is more deleterious than deleting the gene encoding the downstream transcription factor, suggesting that additional branches of the pathway emanating from IreA contribute to the ER stress response independently of HacA (Fig. 1A). To further examine the possibility of more complexity in the pathway, we focused in this study on the RNase domain, incorporating both small-molecule and genetic inhibition approaches.

**FIG 2:**
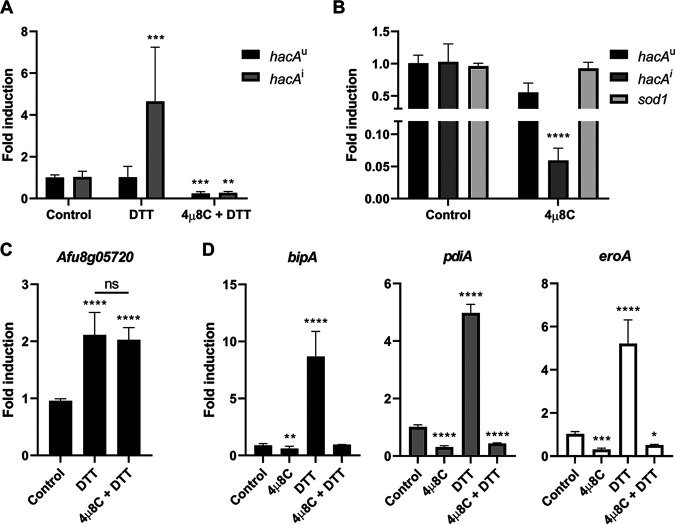
The human IRE1 inhibitor 4μ8C blocks the induction of the canonical *hacA^u^*-*hacA^i^* pathway in A. fumigatus. (A) RT-qPCR analysis of *hacA^u^* and *hacA^i^* mRNA levels during acute ER stress induced by 1 mM DTT for 1 h in the presence or absence of 10 mg/liter 4μ8C. (B) RT-qPCR analysis of *hacA^u^*, *hacA^i^*, and *sod1* mRNA levels in the presence of 10 mg/liter 4μ8C. (C) RT-qPCR analysis of the expression of the UPR-independent gene Afu8g05720 in the presence of 1 mM DTT and 4μ8C. (D) RT-qPCR analysis of UPR target gene expression (*bipA*, *pdiA*, and *eroA*) in the presence of 10 mg/liter 4μ8C and/or 1 mM DTT. Values represent the mean ± SD of results from three biological replicates per strain and condition (ns, not significant; *, *P* < 0.05; **, *P* < 0.01; ***, *P* < 0.001; ****, *P* < 0.0001 [one-way ANOVA with Dunnett’s *post hoc* test for panels A, B, and D or Tukey’s *post hoc* test for panel C]).

The synthetic coumarin derivative 8-formyl-7-hydroxy-4-methylcoumarin (abbreviated as 4μ8C) was initially identified in a high-throughput screen for selective inhibitors of the human IRE1 RNase (30). The molecule forms a Schiff base with a lysine residue in the active site of the RNase domain, which prevents IRE1 from splicing its target mRNA (*XBP1^u^* in humans). Since the homologous lysine is conserved in A. fumigatus IreA (Fig. 1B), we hypothesized that 4μ8C could be used to modulate the processing of *hacA^u^* mRNA into its activated form, *hacA^i^* (Fig. 1A). To test this, we used quantitative reverse transcription-PCR (RT-qPCR) analysis to monitor the induction of *hacA^i^* levels during acute ER stress. Dithiothreitol (DTT) is the most widely used approach to experimentally induce ER stress because it reduces the disulfide bridges that stabilize many proteins in the secretory pathway, resulting in a rapid increase in the level of unfolded proteins that activate the IRE1/IreA sensor (32). Consistent with previous reports (33), DTT treatment of A. fumigatus hyphae induced accumulation of *hacA^i^* mRNA, reflecting activation of the IreA RNase domain (Fig. 2A). Since the *hacA* gene promoter is a target of the HacA transcription factor (34), this surge in *hacA^i^* mRNA levels in the presence of DTT is due to a positive feedback loop that involves three linked events: first, initial activation of the IreA RNase converts basally expressed *hacA^u^* mRNA into *hacA^i^*; second, translation of *hacA^i^* mRNA into the HacA transcription factor increases transcription from the *hacA* gene promoter itself, thereby replenishing the pool of *hacA^u^* mRNA; and third, splicing of *hacA^u^* into *hacA^i^* continues as long as the IreA RNase remains activated (5, 35). We found that incorporation of 4μ8C into the medium prevented DTT from triggering an increase in *hacA^i^* mRNA abundance, demonstrating that the canonical UPR is not responsive to an acute ER stress stimulus in the presence of this compound. The lowest concentration for blocking the UPR under these conditions was determined to be 10 mg/liter (see Fig. S1 in the supplemental material), so this concentration was used for all subsequent experiments in this study.

10.1128/mSphere.00879-20.1 FIG S1 A minimal concentration of 4μ8C is required to block induction of the UPR. RT-qPCR analysis of mRNA levels from the UPR target genes *bipA*, *pdiA*, and *eroA* in liquid AMM at 37°C and 200 rpm in the A. fumigatus strain KU80. Cultures were simultaneously treated with DTT and two different concentrations of 4μ8C for 1 h prior to harvest. Data represent mean values of three technical replicates ± SD from one representative experiment. The experiment was repeated once with similar results. Download FIG S1, TIF file, 0.2 MB. Copyright © 2020 Guirao-Abad et al. 2020 Guirao-Abad et al. This content is distributed under the terms of the Creative Commons Attribution 4.0 International license.

We have previously reported that low levels of *hacA^i^* are also detectable in the absence of added ER stress, representing a basal UPR that functions to buffer fluctuations in protein folding that arise during normal vegetative growth (5). We found that treatment with 4μ8C also reduced the steady-state levels of *hacA^i^* mRNA under these conditions (Fig. 2B), consistent with the ability of the compound to impair basal IreA activation. In contrast, expression of the *sod1* gene, which does not depend on the UPR for its expression (36), was unaffected by 4μ8C treatment (Fig. 2B), suggesting that downregulation of *hacA^i^* mRNA levels by 4μ8C is not due to a general downregulation of transcription. Moreover, since the effects of DTT on cell physiology are not limited to ER protein folding (37, 38), we also compared the effects of 4μ8C on the expression of Afu8g05720, representing a gene that we have previously shown to be induced by DTT independently of the UPR (5). As shown in Fig. 2C, 4μ8C did not block the induction of this gene by DTT, illustrating specificity of 4μ8C treatment for the inhibition of UPR target genes.

Since 4μ8C effectively blocks *hacA^i^* induction during acute ER stress (Fig. 2A), the compound should also prevent the upregulation of UPR target genes that are under the control of the HacA transcription factor. To test this, we compared the expression levels of three genes that are established UPR targets across multiple species: the Hsp70 chaperone gene *bipA*, the protein disulfide isomerase gene *pdiA*, and the oxidoreductase gene *eroA* (34, 39). As expected, all three of these UPR markers were strongly induced by DTT in A. fumigatus (Fig. 2D). However, no induction was detected in the presence of 4μ8C, consistent with the ability of this compound to prevent the accumulation of *hacA^i^* mRNA (Fig. 2D). The compound also blocked the ability of DTT to induce the expression of *srcA* and *pmrA* (Fig. S2A), encoding ER/Golgi P-type Ca^2+^ ATPases that we have recently identified as targets of the UPR in A. fumigatus (33). Interestingly, we found that a Δ*srcA*/Δ*pmrA* double deletion mutant was hypersensitive to the effects of 4μ8C relative to its parental strain (Fig. S2B). Since the absence of these Ca^2+^ ATPases exacerbates ER stress (33), the increased sensitivity of this mutant to 4μ8C is likely to be due to the ability of the compound to prevent the canonical UPR from mounting an adaptive response to low Ca^2+^ levels in the secretory pathway caused by the absence of these Ca^2+^ pumps. We conclude that 4μ8C is able to enter the fungus and reach the IreA sensor, thereby inhibiting the RNase activity that is required for *hacA^u^* mRNA processing and downstream activation of UPR target genes.

10.1128/mSphere.00879-20.2 FIG S2 The compound 4μ8C blocks the transcriptional upregulation of genes encoding P-type Ca^2+^-ATPases during ER stress. (A) Fold change in the expression levels of the *srcA* and *pmrA* genes by RT-qPCR during acute ER stress induced by 1 mM DTT for 1 h in the presence or absence of 10 mg/liter 4μ8C. Values represent the mean ± SD of the results from three technical replicates per strain and condition. (B) The Δ*srcA*/Δ*pmrA* mutant is hypersensitive to 4μ8C. Serial dilutions of conidia from the indicated strains were spotted onto AMM plates containing 4μ8C and incubated for 72 h at 37°C. Download FIG S2, TIF file, 1.3 MB. Copyright © 2020 Guirao-Abad et al. 2020 Guirao-Abad et al. This content is distributed under the terms of the Creative Commons Attribution 4.0 International license.

### 4μ8C impairs growth on a polymeric protein substrate.

**FIG 3:**
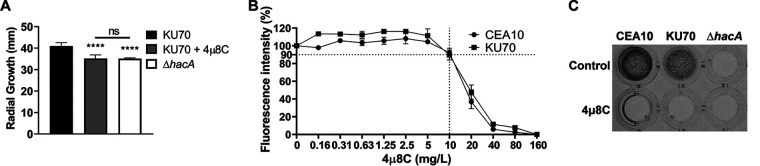
Growth on a complex protein polymer is impaired in the presence of 4μ8C. (A) Conidia were spot inoculated onto the center of plates containing AMM in the presence/absence of 10 mg/liter 4μ8C, and colony diameter was measured after 6 days at 37°C. (B) Overnight cultures in liquid AMM at 37°C were treated with 4μ8C at the indicated concentrations for 1 h, and metabolic activity was determined using resazurin. (C) Conidia from the indicated strains were inoculated onto the surface of a collagen gel matrix containing 10 mg/liter 4μ8C. Images of mycelial growth were captured after 72 h at 37°C. Bars show mean values ± SD of the results from three biological replicates per strain and condition (****, *P* < 0.0001; ns, not significant [one-way ANOVA with Tukey’s *post hoc* test]).

Mutants of A. fumigatus that harbor deletions of the *hacA* gene grow relatively well on medium containing simple carbon and nitrogen sources (3). Similarly, we found that the growth rate of the A. fumigatus parental strain KU70 on solid minimal medium supplemented with 4μ8C was only slightly reduced, comparable in magnitude to the mild growth inhibition displayed by a KU70-derived mutant lacking the *hacA* gene (Fig. 3A). In liquid culture, both KU70 and the unmodified wild-type strain CEA10 showed only a 10% reduction in metabolic activity in the presence of 4μ8C (Fig. 3B), making it unlikely that the inhibitory effect of 4μ8C on UPR gene expression is due to broad inhibition of fungal metabolism. In addition, although a slight reduction in spore germination rate was observed in the presence of 4μ8C, the total biomass accumulation after 24 h of incubation in liquid culture was indistinguishable in the presence or absence of the compound (Fig. S3). We conclude that pharmacological inhibition of the IreA RNase domain has minimal growth-impairing effects on A. fumigatus, consistent with the relatively normal growth characteristics of the Δ*hacA* mutant in the absence of stress.

10.1128/mSphere.00879-20.3 FIG S3 The compound 4μ8C delays germination but has minimal effects on total biomass formation. Microscopic analysis of the parental strain KU70 (A) and the unmodified wild-type isolate CEA10 (B) in the presence of 4μ8C. Scale bar represents 20 μm. (C) Quantification of the germination rate of CEA10 cultures treated with 4μ8C for 16 h. Values represent the mean ± SD of four biological samples per condition. (D) Effect of 4μ8C on biomass formation in liquid culture. The mycelial dry weight was determined from overnight cultures of CEA10 inoculated at 1 × 10^6^ conidia/ml and incubated for 24 h at 37°C. Values represent the mean ± SD of four biological samples per condition (****, *P* < 0.0001 [one-way ANOVA with Dunnett’s *post hoc* test]). Download FIG S3, TIF file, 2.5 MB. Copyright © 2020 Guirao-Abad et al. 2020 Guirao-Abad et al. This content is distributed under the terms of the Creative Commons Attribution 4.0 International license.

The ability of 4μ8C to block the induction of *hacA^i^* mRNA and downstream UPR target gene activation under ER stress predicted that treatment with this compound would have similar effects as deleting the *hacA* gene under conditions that require UPR activity. Filamentous fungi that lack the *hacA* gene grow poorly on polymeric substrates because their secretory pathways are unable to meet the demand for hydrolytic enzyme secretion (3, 9, 40, 41). To determine how 4μ8C would impact utilization of a complex protein substrate, conidia were inoculated into a solution of type I collagen as the only source of carbon and nitrogen. Although both KU70 and CEA10 isolates grew well on this medium, the inclusion of 4μ8C strongly impaired growth (Fig. 3C). The Δ*hacA* mutant also grew poorly on this substrate, indicating that loss of the canonical UPR, either by genetic deletion of *hacA* or by treatment with 4μ8C, impairs the ability to grow on a protein substrate that requires enzymatic breakdown by secreted hydrolases prior to absorption.

### 4μ8C increases sensitivity to the antimicrobial agents carvacrol and hygromycin.

**FIG 4:**
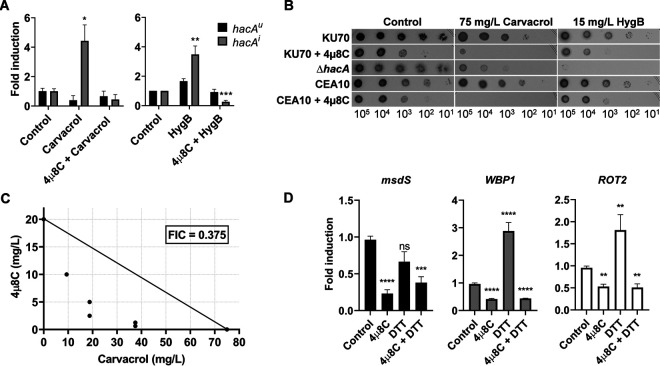
4μ8C increases susceptibility to carvacrol and hygromycin. (A) Fold change in the expression of *hacA^u^* and *hacA^i^* mRNAs by RT-qPCR after treatment for 1 h with 10 mg/liter 4μ8C and/or 75 mg/liter carvacrol or 60 mg/liter hygromycin B (HygB) (strain CEA10). (B) Serial dilutions of conidia from the indicated strains were spotted onto AMM plates containing carvacrol or hygromycin B in the presence or absence of 10 mg/liter 4μ8C and incubated for 48 h at 37°C. A Δ*hacA* mutant that lacks the HygB resistance marker was used in this assay (strain 467). (C) Isobolographic analysis of the synergism between 4μ8C and carvacrol against the wild-type strain CEA10. Solid circles represent the MICs displayed by different combinations of the drugs. (D) Fold change in the expression levels of the glycosylation-related genes *WBP1* (Afu5g08970), *ROT2* (Afu5g03500), and *msdS* (Afu1g14560) in the presence or absence of 10 mg/liter 4μ8C. Values represent the mean ± SD of the results from three biological replicates per strain and condition (*, *P* < 0.05; **, *P* < 0.01; ***, *P* < 0.001; ****, *P* < 0.0001; ns, not significant [one-way ANOVA with Dunnett’s *post hoc* test]).

The inability of A. fumigatus to activate the canonical UPR in the presence of 4μ8C suggests that the fungus would be hypersensitive to a drug that causes ER stress. To test this, the effects of 4μ8C on growth were compared in the presence of carvacrol, a plant-derived compound that disrupts ER homeostasis in Candida albicans and triggers UPR intervention (42). As shown in Fig. 4A, carvacrol induced accumulation of the *hacA^i^* mRNA in the CEA10 strain, confirming that this compound has similar adverse effects on ER homeostasis in A. fumigatus as it does in C. albicans. In addition, the Δ*hacA* mutant was hypersensitive to carvacrol relative to its parental strain KU70, indicating a role for the canonical UPR in protecting against carvacrol-induced ER stress (Fig. 4B). We found that treatment with 4μ8C prevented carvacrol-induced *hacA^i^* accumulation (Fig. 4A), which was associated with increased susceptibility to carvacrol in both the KU70 and CEA10 backgrounds (Fig. 4B). An isobolographic analysis (43) in liquid culture confirmed that the combination of 4μ8C and carvacrol treatment had synergistic toxicity toward the fungus (Fig. 4C). We conclude that preventing the canonical UPR from responding to carvacrol-induced ER stress, either by chemical treatment with 4μ8C or by genetic deletion of *hacA*, is toxic to A. fumigatus.

Other plant-derived compounds that are structurally related to carvacrol have been shown to enhance the toxicity of drugs that target translation fidelity, such as aminoglycosides (44). Aminoglycosides are a therapeutically important class of antimicrobials that reduce the discrimination between cognate and near-cognate tRNAs, causing the incorporation of incorrect amino acids that promotes the accumulation of toxic protein aggregates (45). As shown in Fig. 4A, the aminoglycoside hygromycin B (HygB) induced *hacA^i^* in A. fumigatus, which was blocked by treatment with 4μ8C (Fig. 4A), suggesting a role for the UPR in countering protein folding stress associated with HygB-induced translational errors. In support of this, we found that the Δ*hacA* mutant was hypersensitive to HygB and that treatment of either KU70 or CEA10 strains with 4μ8C increased sensitivity to this compound (Fig. 4B). An alternative explanation for this increased HygB susceptibility stems from the observation that yeast glycosylation mutants are hypersensitive to this aminoglycoside, a phenomenon that is attributed to one or more HygB resistance proteins that require glycosylation for optimal activity (46). Our previous demonstration that the Δ*hacA* mutant is deficient in the expression of genes involved in N-glycan biosynthesis (5) would be consistent with a glycosylation defect that enhances HygB sensitivity. This predicts that UPR inhibition by 4μ8C would also reduce the expression of glycosylation-related genes. To test this, we compared the expression levels of glycosylation genes that were previously shown to be downregulated in the Δ*hacA* mutant (5): *msdS* (encoding an α-mannosidase) (47), the DTT-inducible gene Afu5g08970 (*WBP1* in Saccharomyces cerevisiae, an oligosaccharyl transferase gene), and Afu5g03500 (*ROT2* in S. cerevisiae, a glucosidase I gene). Similarly to Δ*hacA* (Fig. S4), all three genes were downregulated in A. fumigatus after treatment with 4μ8C as demonstrated by RT-qPCR analysis (Fig. 4D). As expected, the induction of *WBP1* and *ROT1* upon ER stress with 1 mM DTT was blocked in the presence of the inhibitor (Fig. 4D). We conclude that the canonical UPR is an integral part of the cellular response to HygB in A. fumigatus and that preventing UPR activation, either by treatment with 4μ8C or by genetic deletion of *hacA*, enhances the toxicity of this aminoglycoside against the fungus.

10.1128/mSphere.00879-20.4 FIG S4 Glycosylation-related gene expression is reduced by *hacA* deletion or by treatment with 4μ8C. RT-qPCR analysis of the expression levels of the glycosylation-related genes *msdS* (Afu1g14560), *WBP1* (Afu5g08970), and *ROT2* (Afu5g03500) in the presence or absence of 4μ8C. Values represent the mean ± SD of the results from three technical replicates per strain and condition. Download FIG S4, TIF file, 0.2 MB. Copyright © 2020 Guirao-Abad et al. 2020 Guirao-Abad et al. This content is distributed under the terms of the Creative Commons Attribution 4.0 International license.

### 4μ8C reveals unexpected complexity in the canonical UPR pathway.

**FIG 5:**
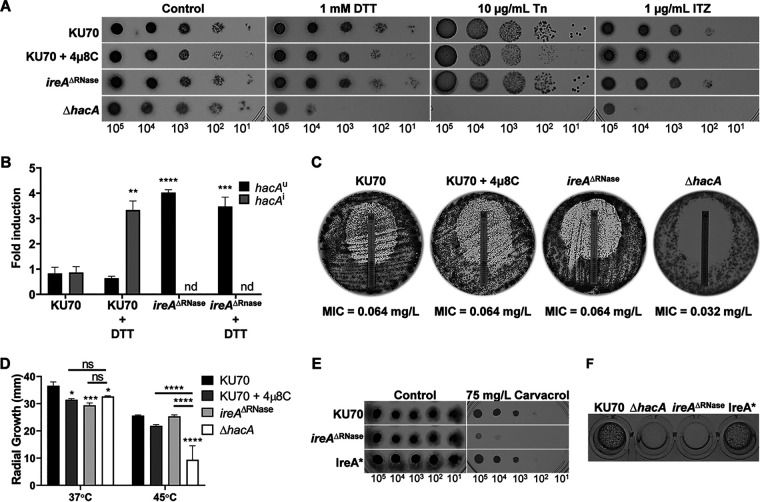
The compound 4μ8C reveals additional complexity in the canonical UPR pathway. (A) Serial dilutions of conidia from the indicated strains were spotted onto AMM plates containing DTT, tunicamycin (Tn), or itraconazole (ITZ) in the presence or absence of 10 mg/liter 4μ8C and incubated for 48 h at 37°C. (B) Fold change in the expression of *hacA^u^* and *hacA^i^* mRNAs by RT-qPCR after treatment of the indicated strains for 1 h with 1 mM DTT. Cultures inoculated at 10^6^ conidia/ml were grown in AMM for 16 h at 37°C. (C) The caspofungin sensitivities of the *ireA*^ΔRNase^ and Δ*hacA* mutants were compared to that of the parental strain KU70 in the presence or absence of 4μ8C using the MIC test strip method. (D) Colony diameters on minimal medium at 37°C and 45°C. Conidia from the indicated strains were spot inoculated onto the center of plates containing AMM, and colony diameter was measured after 6 days at 37°C. (E) Serial dilutions of conidia from the indicated strains were spotted onto AMM plates containing carvacrol and incubated for 72 h at 37°C. (F) Conidia from the indicated strains were inoculated onto the surface of a collagen gel matrix, and mycelial growth was photographed after 72 h at 37°C. Values in panels B and D represent the mean ± SD of the results from three biological replicates per strain and condition (*, *P* < 0.05; **, *P* < 0.01; ***, *P* < 0.001; ****, *P* < 0.0001; nd, not detected; ns, not significant [one-way ANOVA with Dunnett’s {B} or Tukey’s {D} *post hoc* test]).

The Δ*hacA* mutant of A. fumigatus exhibits increased sensitivity to both the triazole and echinocandin classes of antifungals, as well as to the ER stress agents DTT and tunicamycin (3). Although blocking *hacA^i^* mRNA induction by 4μ8C treatment increased carvacrol and hygromycin B sensitivity, we were surprised to find that the compound had minimal effects on the sensitivity of the fungus to DTT, tunicamycin, or itraconazole (Fig. 5A). A fundamental difference between the Δ*hacA* mutant and chemical inhibition of *hacA^u^* processing is that the Δ*hacA* mutant lacks both *hacA^u^* and *hacA^i^* mRNAs due to the absence of the *hacA* gene, whereas the 4μ8C-treated organism still expresses *hacA^u^* (Fig. 2B). This raises the possibility that residual *hacA^i^* levels in cultures treated with 4μ8C are sufficient to provide some protection against ER stress. We therefore examined a mutant that harbors an inactivating deletion in the RNase domain of IreA but still retains the *hacA* gene (*ireA*^ΔRNase^). As observed with 4μ8C treatment, no *hacA^i^* mRNA could be detected by RT-qPCR in the *ireA*^ΔRNase^ mutant, even in the presence of a strong ER stress stimulus, confirming that this strain is incapable of mounting a canonical UPR response (Fig. 5B). The levels of *hacA^u^* were notably elevated in the *ireA*^ΔRNase^ mutant (Fig. 5B), consistent with a failure to process basally expressed *hacA^u^* mRNA into *hacA^i^*. Interestingly, and similar to what we observed with 4μ8C treatment, the *ireA*^ΔRNase^ mutant showed no hypersensitivity to DTT, tunicamycin, or itraconazole (Fig. 5A).

We have previously shown that the Δ*hacA* mutant has a lower MIC to caspofungin using an MIC test strip and that the normally fungistatic effects of caspofungin toward A. fumigatus became fungicidal in the absence of *hacA* (Fig. 5C) (3). In contrast, caspofungin remained fungistatic to the *ireA*^ΔRNase^ mutant and the 4μ8C-treated parental strain, with no decrease in the MIC (Fig. 5C). In addition, we found that neither the *ireA*^ΔRNase^ mutant nor treatment with 4μ8C increased thermal stress sensitivity as previously reported for the Δ*hacA* mutant (Fig. 5D) (3). However, the *ireA*^ΔRNase^ mutant was hypersensitive to carvacrol and grew poorly on a collagen substrate (Fig. 5E and F), similar to the effects of 4μ8C on the control strain (Fig. 3C and Fig. 4B). Together, these findings indicate that blocking *hacA^i^* accumulation under conditions of ER stress by either a chemical or genetic approach is less detrimental to the fungus than the complete absence of the *hacA* gene, suggesting that there is additional complexity in the pathway that involves functions for the unspliced *hacA^u^* mRNA that are distinct from the spliced mRNA.

## DISCUSSION

Selective inhibitors of the kinase and/or RNase domains of human IRE1 are under development for the purpose of creating toxic levels of unfolded proteins in human tumor cells, particularly when used in combination with drugs that exacerbate ER stress (18, 19, 48, 49). These compounds are well tolerated in animal models and have been associated with favorable therapeutic outcomes (21–24, 50). However, their effects on a fungal pathogen have not been reported. Here, we demonstrate that the human IRE1 RNase inhibitor 4μ8C impaired the ability of A. fumigatus to maintain basal levels of *hacA^i^* mRNA under vegetative growth conditions, in addition to preventing the accumulation of *hacA^i^* under conditions of acute ER stress induced by treatment with DTT or the natural product carvacrol. This correlated with reduced expression levels of known UPR target genes, reflecting inhibition of the canonical UPR. STF-083010 is another inhibitor of the human IRE1 RNase and is structurally related to 4μ8C (21). We found that this compound also impaired *hacA^i^* mRNA induction and sensitized the fungus to carvacrol-induced ER stress (see Fig. S5 in the supplemental material), providing additional support for the notion that disabling the fungal UPR is achievable by small-molecule inhibition. However, in contrast to 4μ8C, STF-083010 was only partially effective, so it was not pursued further in this study. Our data on 4μ8C provide the first validation of small-molecule inhibition of the fungal UPR in A. fumigatus, which could provide a valuable tool for future studies into how this stress response pathway integrates with other cellular circuitry to coordinate adaptive responses in fungi. It is also worth mentioning that UPR activity impacts one of the bottlenecks that limit the secretion of proteins by filamentous fungi used in the biotechnology industry (51), suggesting that 4μ8C could have broader utility as a way to understand how the UPR affects industrial secretion processes.

10.1128/mSphere.00879-20.5 FIG S5 The human IRE1 inhibitor STF-083010 partially impairs the fungal UPR. (A) Treatment with STF-083010 increases sensitivity to carvacrol. Serial dilutions of conidia from the indicated strains were spotted onto AMM plates containing carvacrol and/or STF-083010 and incubated for 48 h at 37°C. (B) Fold change in the expression of *hacA^u^* and *hacA^i^* mRNAs by RT-qPCR after overnight cultures (20 h at 37°C) were treated for 1 h with DTT in the presence or absence of STF-083010 (strain CEA10). (C) Treatment with STF-083010 partially blocks DTT induction of the UPR target gene *bipA*. Fold change in the expression of *bipA* mRNA by RT-qPCR after overnight cultures (20 h at 37°C) were treated for 1 h with DTT in the presence or absence of STF-083010 (strain CEA10). Values represent the mean ± SD of the results from three technical replicates per strain and condition. Download FIG S5, TIF file, 0.4 MB. Copyright © 2020 Guirao-Abad et al. 2020 Guirao-Abad et al. This content is distributed under the terms of the Creative Commons Attribution 4.0 International license.

It is well known that filamentous fungi possess a high capacity to secrete large quantities of hydrolytic enzymes that allow them to break down complex substrates (52). Minimal medium that is optimized for the growth of A. fumigatus contains simple forms of carbon and nitrogen that are readily assimilated by the fungus. Treatment with 4μ8C had only a minor inhibitory effect on growth in this medium, similar to the mild growth impairment displayed by a Δ*hacA* mutant under the same conditions (Fig. 3). In contrast, either deletion of *hacA* or treatment with 4μ8C severely impaired the ability of A. fumigatus to grow on a collagen substrate. This is consistent with the importance of the UPR in supporting the accurate folding of secreted hydrolytic enzymes that are necessary to break down complex biopolymers (Fig. 3C).

Carvacrol is an essential oil from the oregano plant with *in vitro* antifungal activity against a variety of fungal species (42, 53–56) and has been shown to have potential *in vivo* application as a prophylactic agent for the prevention of avian aspergillosis (57). Although the precise molecular target of carvacrol is unknown, its ability to disrupt ER morphology and induce a UPR transcriptional signature in C. albicans clearly indicates that it causes acute ER stress (42). We found that carvacrol also induced the UPR in A. fumigatus, which could be blocked by treatment with 4μ8C (Fig. 4A). The two compounds showed synergistic toxicity against A. fumigatus, suggesting that 4μ8C prevents the accumulation of *hacA^i^* mRNA needed to counteract the toxic effects of carvacrol on ER homeostasis (Fig. 4B and C). We also found that 4μ8C increased the susceptibility of C. albicans to carvacrol, suggesting a conserved mechanism of carvacrol between species (Fig. S6). Interestingly, our findings also revealed enhanced toxicity of hygromycin B against A. fumigatus in the presence of 4μ8C. This is likely to be due, in part, to the ability of 4μ8C to prevent the canonical UPR from adequately responding to the ER stress caused by hygromycin-induced errors in protein translation (Fig. 4A). In addition, the adverse effects of 4μ8C on the expression of glycosylation-related genes (Fig. 4D) would be expected to impair glycosylation-assisted protein folding, as well as impair glycosylation-dependent hygromycin B resistance mechanisms that have been well described from studies on yeast glycosylation mutants (46). We conclude that 4μ8C-induced hypersensitivity to hygromycin B is likely to be multifactorial but mediated in part by UPR signaling through the canonical HacA^i^-directed pathway.

10.1128/mSphere.00879-20.6 FIG S6 Susceptibility to carvacrol is enhanced by 4μ8C treatment in C. albicans. Serial 10-fold dilutions of C. albicans yeast (ATCC 18804) were spotted onto plates of yeast extract-peptone-dextrose (YPD) agar containing 4μ8C in the presence or absence of carvacrol and incubated at 37°C for 24 h. Download FIG S6, TIF file, 0.5 MB. Copyright © 2020 Guirao-Abad et al. 2020 Guirao-Abad et al. This content is distributed under the terms of the Creative Commons Attribution 4.0 International license.

Despite the existence of two overlapping reading frames in all species homologs of the mRNA encoding the UPR transcription factor, it is widely assumed that the spliced mRNA is the most relevant to ER homeostasis because it translates a bZIP transcription factor involved in UPR target gene expression. Interestingly, a phylogenetic analysis of vertebrate homologs of this mRNA has indicated that the evolution of the two overlapping reading frames argues for functionality of the unspliced transcript protein (58). Recent studies in human cells have shown that one function of the protein specified by the unspliced transcript is to target the nascent protein-mRNA-ribosome complex to the ER membrane via the signal recognition particle (SRP) pathway (59–62). If the sole purpose of the protein encoded by the unspliced mRNA in A. fumigatus is bringing the *hacA^u^* mRNA in the proximity of the IreA RNase in order to optimize splicing into *hacA^i^*, one would predict that preventing *hacA^i^* accumulation by treatment with 4μ8C or by IreA RNase domain mutation would have the same effects as deleting the *hacA* gene (Fig. 1A). However, our data show that the effects of deleting *hacA* are broader in scope relative to chemical or genetic RNase inhibition (Fig. 5), suggesting that there is unexplained functionality of the unspliced *hacA^u^* mRNA that goes beyond serving as the precursor for *hacA^i^*. In Cryptococcus neoformans, the majority of the phenotypes associated with *ire1* deletion could be complemented with the spliced transcription factor gene but not by the unspliced version (10). However, in that study complementation was performed in the complete absence of Ire1; in our study Ire1 was still present but rendered incapable of inducing the canonical pathway due to chemical or genetic inhibition of the Ire1 RNase. Ire1 is also present in the Δ*hacA* mutant of A. fumigatus, but the phenotype of that mutant is more severe than the phenotype of canonical UPR inhibition using 4μ8c treatment or RNase mutation, suggesting that beneficial effects of *hacA^u^* are apparent when the Ire1 sensor is present. This raises the possibility that the unspliced protein augments noncanonical functions of Ire1, which would be consistent with evidence that it is targeted to the ER membrane (59–62). Unique functions for the unspliced protein have also been proposed in human cells (63, 64), as well as in Aspergillus oryzae, where mutants that constitutively express only the spliced form exhibit a transcriptional profile that is different from that of a *hacA* deletion strain (65). The existence of additional noncanonical branches in the UPR that contribute to ER homeostasis is also consistent with studies showing that the UPR is capable of customizing the regulation of the target gene expression contingent upon the nature and scope of the stress that a cell encounters, rather than acting as a simple “on-off” switch (66, 67). Experiments to explore the mechanisms involved in A. fumigatus are under way and are expected to further unravel the complexity of the UPR in the biology and virulence of this clinically important fungal pathogen.

## MATERIALS AND METHODS

### Reagents.

Aliquots of 4μ8C (EMD Millipore; 412512) at 25 mg/ml were prepared in dimethyl sulfoxide (DMSO) and stored at −20°C until use. Dithiothreitol (DTT; Thermo Scientific; R0862) was dissolved in water at 1 M prior to use. Tunicamycin (Cayman Chemical; 11445) and itraconazole (Sigma; 16657) were dissolved in DMSO at 10 mg/liter and stored until use at −20°C or −80°C, respectively. Hygromycin B (RPI; H75020) was dissolved in water at 100 mg/ml and stored at −80°C prior to use. A 6.4 M stock solution of carvacrol (Sigma-Aldrich; W224511) was diluted to 0.64 M (96.74 g/liter) in DMSO prior to immediate use.

### Strains and culture conditions.

The A. fumigatus strains used in this study are summarized in Table S1 in the supplemental material. Conidia were harvested from cultures grown on OSM plates (*Aspergillus* minimal medium [AMM] osmotically stabilized with 1.2 M sorbitol). Unless otherwise specified, all experiments were performed in AMM: 1% (wt/vol) d-glucose, 1% (vol/vol) NH_4_ tartrate, and 2% (vol/vol) salt solution (2.6% [wt/vol] KCl, 2.6% [wt/vol] MgSO_4_ heptahydrate, 7.6% [wt/vol] KH_2_PO_4_, 5% [vol/vol] trace-element solution). Colony diameters were determined by spotting 5 × 10^3^ conidia onto the center of a 100-mm plate containing AMM + 0.8% (wt/vol) agarose (UltraPure agarose; Invitrogen) with or without 4μ8C, and colony diameters were measured after 6 days. For analysis of stress sensitivity, serial dilutions of conidia (10^5^ to 10^1^ conidia in a 5-μl volume) were spotted onto AMM plates supplemented with 0.8% agarose and the compounds of interest. Plates were incubated at 37°C and photographed after 48 h. For analysis of germination rates, a total of 1 × 10^3^ spores were inoculated into liquid AMM containing the indicated concentrations of 4μ8C and incubated at 37°C. Controls with DMSO were run in parallel. The number of germinated conidia was then quantified microscopically (90 to 183 cells counted per condition). For analysis of mycelial biomass, liquid AMM was inoculated with CEA10 conidia at a concentration of 1 × 10^6^ conidia/ml and incubated for 24 h at 37°C. The mycelium was then dried and weighed. Growth on a collagen substrate was performed by diluting a 10× stock solution of *Aspergillus* minimal salts to 1× with a 5-mg/liter solution of type l collagen as the sole source of carbon and nitrogen (Sigma; C3867; 5 mg/liter), and inoculating 100 conidia into the surface of the gel in a 96-well plate. Plates were photographed after 72 h at 37°C. Control cultures in liquid AMM were prepared in parallel.

10.1128/mSphere.00879-20.7 TABLE S1 Strains of A. fumigatus used in this study. Download Table S1, DOCX file, 0.02 MB. Copyright © 2020 Guirao-Abad et al. 2020 Guirao-Abad et al. This content is distributed under the terms of the Creative Commons Attribution 4.0 International license.

### RNA extraction and RT-qPCR.

Cultures were inoculated with 1 × 10^6^ conidia/ml in 50 ml of AMM and incubated at 37°C and 200 rpm. After 16 h of incubation, cultures were treated for 1 h with the indicated compounds prior to RNA isolation. RNA was isolated using the RNAzol RT column kit according to the manufacturer’s instructions (Molecular Research Center, Inc.). The digestion of genomic DNA and synthesis of cDNA were performed using RNase-free DNase (Roche) and iScript Reverse Transcription Supermix for RT-qPCR (Bio-Rad). The RT-qPCR was performed in a StepOne real-time PCR system (Applied Biosystems) using 1 μg of template per well and iTaq Universal SYBR Green Supermix (Bio-Rad). The primers used in the reaction mix are summarized in Table S2, and their final concentration was 500 nM, with the exception of 200 nM for the housekeeping gene (18S rRNA). Primer efficacy was evaluated using a standard curve. The specificity of the *hacA^u^* and *hacA^i^* reverse primers for distinguishing between the unspliced and spliced versions of the *hacA* mRNA was confirmed by RT-qPCR using the TaqMan probe and primers listed in Table S2. The final concentration for probes and primers was 250 nM and 500 nM, respectively (including the housekeeping gene).The reaction was performed with the *Ex Taq* master mix (TaKaRa), using the same cycle parameters described above for RT-qPCR. Since expression results obtained with SYBR green were similar to those obtained with a TaqMan probe, the SYBR green method was employed for the results presented in this study, using the same primer concentrations used for TaqMan. For *hacA^u^* and *hacA^i^* mRNA detection, the cycle conditions were 20 s at 95°C, 40 cycles of 3 s at 95°C, and 20 s at 66°C. For detection of other genes, the cycle conditions were 20 s at 95°C, 40 cycles of 3 s at 95°C, and 30 s at 60°C. The melting curve was monitored to verify the specificity of the amplification reaction. The 18S rRNA was used as a housekeeping gene. Fold change in mRNA levels was determined in comparison to untreated cultures.

10.1128/mSphere.00879-20.8 TABLE S2 Oligonucleotides (primers and probes) used for gene expression analyses. Download Table S2, DOCX file, 0.02 MB. Copyright © 2020 Guirao-Abad et al. 2020 Guirao-Abad et al. This content is distributed under the terms of the Creative Commons Attribution 4.0 International license.

### Analysis of metabolic activity and antifungal susceptibility.

For analysis of metabolic activity, conidia were inoculated at a concentration of 1 × 10^6^ conidia/ml in 150 μl of liquid AMM in a 96-well plate and incubated for 16 h at 37°C. The plates were washed three times, and the indicated concentrations of 4μ8C were added to the plates before incubating for an additional hour at 37°C. The final concentration of DMSO was 0.5% (vol/vol) in each well, including the growth controls. The cultures were then washed three times, and the medium was replaced with AMM containing 0.02 mg/ml of the oxidation-reduction metabolic indicator resazurin (14322; Cayman Chemicals). After incubating for 1 h at 37°C, the fluorescence was measured (excitation, 535 nm; emission, 590 nm) in a microplate reader (Synergy H1; BioTek). For analysis of synergy between carvacrol and 4μ8C, conidia were inoculated into liquid AMM containing 0.02 mg/ml of resazurin at a concentration of 2.5 × 10^4^ conidia/ml. Serial 2-fold dilutions of 4μ8C were then dispensed from column 2 in the 96-well plate to column 9. Additionally, 2-fold dilutions of carvacrol were dispensed from row A to H into each well containing 4μ8C. Columns 1 and 11 were used as growth and sterility controls, respectively. Columns 10 and 12 were used to display the individual MICs of carvacrol and 4μ8C. The plates were incubated at 37°C for 24 h, after which the fluorescence was measured as described above. The MIC was defined as the lowest concentration required to inhibit 90% of fungal metabolic activity after 24 h of incubation. An isobologram representation was used to determine if the combination of 4μ8C and carvacrol had synergistic activity. The diagonal line in Fig. 4C connects the individual MICs for each compound (shown by the solid circles on the axis lines), and the remaining circles represent the MICs for different combinations of the two drugs. Synergistic, additive, and antagonistic effects are represented by solid circles that fall below the diagonal line, on top of the diagonal line, or above the diagonal line, respectively. The fractional inhibitory concentration (FIC) index was determined as (MIC carvacrol in combination with 4μ8C/MIC carvacrol alone) + (MIC 4μ8C in combination with carvacrol/MIC 4μ8C alone). The interaction between the drugs was defined as synergistic (FIC < 0.5), indifferent (0.5 < FIC ≤ 4.0), or antagonistic (FIC > 4.0). Antifungal susceptibility using the MIC test strip (MTS) method was determined following the instructions of the manufacturer. A concentration of 10^6^ conidia/ml was spread with a sterile cotton swab onto a plate of RPMI 1640 agar with 0.164 M morpholinepropanesulfonic acid (MOPS) and l-glutamine (pH 7.0). RPMI plates containing 4μ8C were run in parallel. After the surface of the medium appeared dry, an MIC test strip containing caspofungin (Liofilchem; 92154) was applied. Plates were incubated for 48 h at 37°C and photographed.

### Statistical analysis.

Statistical analysis was performed using GraphPad Prism version 8 for Windows (GraphPad Software, San Diego, CA, USA). Statistically significant differences were determined using one-way analyses of variance (ANOVA) with Dunnett’s or Tukey’s multiple-comparison tests.

## References

[1] Preissler S, Ron D. 2019. Early events in the endoplasmic reticulum unfolded protein response. Cold Spring Harb Perspect Biol 11:a033894. doi:10.1101/cshperspect.a033894. 30396883 PMC6442202

[2] Hampel M, Jakobi M, Schmitz L, Meyer U, Finkernagel F, Doehlemann G, Heimel K. 2016. Unfolded protein response (UPR) regulator Cib1 controls expression of genes encoding secreted virulence factors in *Ustilago maydis*. PLoS One 11:e0153861. doi:10.1371/journal.pone.0153861. 27093436 PMC4836707

[3] Richie DL, Hartl L, Aimanianda V, Winters MS, Fuller KK, Miley MD, White S, McCarthy JW, Latgé J-P, Feldmesser M, Rhodes JC, Askew DS. 2009. A role for the unfolded protein response (UPR) in virulence and antifungal susceptibility in *Aspergillus fumigatus*. PLoS Pathog 5:e1000258. doi:10.1371/journal.ppat.1000258. 19132084 PMC2606855

[4] Bitencourt TA, Lang EAS, Sanches PR, Peres NTA, Oliveira VM, Fachin AL, Rossi A, Martinez-Rossi NM. 2020. HacA governs virulence traits and adaptive stress responses in *Trichophyton rubrum*. Front Microbiol 11:193. doi:10.3389/fmicb.2020.00193. 32153523 PMC7044415

[5] Feng X, Krishnan K, Richie DL, Aimanianda V, Hartl L, Grahl N, Powers-Fletcher MV, Zhang M, Fuller KK, Nierman WC, Lu LJ, Latgé J-P, Woollett L, Newman SL, Cramer RA, Rhodes JC, Askew DS. 2011. HacA-independent functions of the ER stress sensor IreA synergize with the canonical UPR to influence virulence traits in *Aspergillus fumigatus*. PLoS Pathog 7:e1002330. doi:10.1371/journal.ppat.1002330. 22028661 PMC3197630

[6] Wimalasena TT, Enjalbert B, Guillemette T, Plumridge A, Budge S, Yin Z, Brown AJP, Archer DB. 2008. Impact of the unfolded protein response upon genome-wide expression patterns, and the role of Hac1 in the polarized growth, of *Candida albicans*. Fungal Genet Biol 45:1235–1247. doi:10.1016/j.fgb.2008.06.001. 18602013

[7] Xu D, Jiang B, Ketela T, Lemieux S, Veillette K, Martel N, Davison J, Sillaots S, Trosok S, Bachewich C, Bussey H, Youngman P, Roemer T. 2007. Genome-wide fitness test and mechanism-of-action studies of inhibitory compounds in *Candida albicans*. PLoS Pathog 3:e92. doi:10.1371/journal.ppat.0030092. 17604452 PMC1904411

[8] Miyazaki T, Nakayama H, Nagayoshi Y, Kakeya H, Kohno S. 2013. Dissection of Ire1 functions reveals stress response mechanisms uniquely evolved in *Candida glabrata*. PLoS Pathog 9:e1003160. doi:10.1371/journal.ppat.1003160. 23382685 PMC3561209

[9] Joubert A, Simoneau P, Campion C, Bataillé-Simoneau N, Iacomi-Vasilescu B, Poupard P, François JM, Georgeault S, Sellier E, Guillemette T. 2011. Impact of the unfolded protein response on the pathogenicity of the necrotrophic fungus *Alternaria brassicicola*. Mol Microbiol 79:1305–1324. doi:10.1111/j.1365-2958.2010.07522.x. 21251090

[10] Cheon SA, Jung K-W, Chen Y-L, Heitman J, Bahn Y-S, Kang HA. 2011. Unique evolution of the UPR pathway with a novel bZIP transcription factor, Hxl1, for controlling pathogenicity of *Cryptococcus neoformans*. PLoS Pathog 7:e1002177. doi:10.1371/journal.ppat.1002177. 21852949 PMC3154848

[11] Blankenship JR, Fanning S, Hamaker JJ, Mitchell AP. 2010. An extensive circuitry for cell wall regulation in *Candida albicans*. PLoS Pathog 6:e1000752. doi:10.1371/journal.ppat.1000752. 20140194 PMC2816693

[12] Jung K-W, Lee K-T, Averette AF, Hoy MJ, Everitt J, Heitman J, Bahn Y-S. 2018. Evolutionarily conserved and divergent roles of unfolded protein response (UPR) in the pathogenic *Cryptococcus* species complex. Sci Rep 8:8132. doi:10.1038/s41598-018-26405-5. 29802329 PMC5970146

[13] Calfon M, Zeng H, Urano F, Till JH, Hubbard SR, Harding HP, Clark SG, Ron D. 2002. IRE1 couples endoplasmic reticulum load to secretory capacity by processing the *XBP-1* mRNA. Nature 415:92–96. doi:10.1038/415092a. 11780124

[14] Krishnan K, Askew DS. 2014. Endoplasmic reticulum stress and fungal pathogenesis. Fungal Biol Rev 28:29–35. doi:10.1016/j.fbr.2014.07.001. 25419229 PMC4235150

[15] Cox JS, Shamu CE, Walter P. 1993. Transcriptional induction of genes encoding endoplasmic reticulum resident proteins requires a transmembrane protein kinase. Cell 73:1197–1206. doi:10.1016/0092-8674(93)90648-a. 8513503

[16] Mori K, Ma W, Gething MJ, Sambrook J. 1993. A transmembrane protein with a cdc2+/CDC28-related kinase activity is required for signaling from the ER to the nucleus. Cell 74:743–756. doi:10.1016/0092-8674(93)90521-Q. 8358794

[17] Cox JS, Walter P. 1996. A novel mechanism for regulating activity of a transcription factor that controls the unfolded protein response. Cell 87:391–404. doi:10.1016/s0092-8674(00)81360-4. 8898193

[18] Maly DJ, Papa FR. 2014. Druggable sensors of the unfolded protein response. Nat Chem Biol 10:892–901. doi:10.1038/nchembio.1664. 25325700 PMC4664160

[19] Wang M, Law ME, Castellano RK, Law BK. 2018. The unfolded protein response as a target for anticancer therapeutics. Crit Rev Oncol Hematol 127:66–79. doi:10.1016/j.critrevonc.2018.05.003. 29891114

[20] Koong AC, Chauhan V, Romero-Ramirez L. 2006. Targeting XBP-1 as a novel anti-cancer strategy. Cancer Biol Ther 5:756–759. doi:10.4161/cbt.5.7.2973. 16861911

[21] Papandreou I, Denko NC, Olson M, Van Melckebeke H, Lust S, Tam A, Solow-Cordero DE, Bouley DM, Offner F, Niwa M, Koong AC. 2011. Identification of an Ire1alpha endonuclease specific inhibitor with cytotoxic activity against human multiple myeloma. Blood 117:1311–1314. doi:10.1182/blood-2010-08-303099. 21081713 PMC3056474

[22] Sheng X, Nenseth HZ, Qu S, Kuzu OF, Frahnow T, Simon L, Greene S, Zeng Q, Fazli L, Rennie PS, Mills IG, Danielsen H, Theis F, Patterson JB, Jin Y, Saatcioglu F. 2019. IRE1α-XBP1s pathway promotes prostate cancer by activating c-MYC signaling. Nat Commun 10:323. doi:10.1038/s41467-018-08152-3. 30679434 PMC6345973

[23] Zhao N, Cao J, Xu L, Tang Q, Dobrolecki LE, Lv X, Talukdar M, Lu Y, Wang X, Hu DZ, Shi Q, Xiang Y, Wang Y, Liu X, Bu W, Jiang Y, Li M, Gong Y, Sun Z, Ying H, Yuan B, Lin X, Feng X-H, Hartig SM, Li F, Shen H, Chen Y, Han L, Zeng Q, Patterson JB, Kaipparettu BA, Putluri N, Sicheri F, Rosen JM, Lewis MT, Chen X. 2018. Pharmacological targeting of MYC-regulated IRE1/XBP1 pathway suppresses MYC-driven breast cancer. J Clin Invest 128:1283–1299. doi:10.1172/JCI95873. 29480818 PMC5873887

[24] Tufanli O, Telkoparan Akillilar P, Acosta-Alvear D, Kocaturk B, Onat UI, Hamid SM, Çimen I, Walter P, Weber C, Erbay E. 2017. Targeting IRE1 with small molecules counteracts progression of atherosclerosis. Proc Natl Acad Sci U S A 114:E1395–E1404. doi:10.1073/pnas.1621188114. 28137856 PMC5338400

[25] Volkmann K, Lucas JL, Vuga D, Wang X, Brumm D, Stiles C, Kriebel D, Der-Sarkissian A, Krishnan K, Schweitzer C, Liu Z, Malyankar UM, Chiovitti D, Canny M, Durocher D, Sicheri F, Patterson JB. 2011. Potent and selective inhibitors of the inositol-requiring enzyme 1 endoribonuclease. J Biol Chem 286:12743–12755. doi:10.1074/jbc.M110.199737. 21303903 PMC3069474

[26] Ghosh R, Wang L, Wang ES, Perera BGK, Igbaria A, Morita S, Prado K, Thamsen M, Caswell D, Macias H, Weiberth KF, Gliedt MJ, Alavi MV, Hari SB, Mitra AK, Bhhatarai B, Schürer SC, Snapp EL, Gould DB, German MS, Backes BJ, Maly DJ, Oakes SA, Papa FR. 2014. Allosteric inhibition of the IRE1α RNase preserves cell viability and function during endoplasmic reticulum stress. Cell 158:534–548. doi:10.1016/j.cell.2014.07.002. 25018104 PMC4244221

[27] Tomasio SM, Harding HP, Ron D, Cross BCS, Bond PJ. 2013. Selective inhibition of the unfolded protein response: targeting catalytic sites for Schiff base modification. Mol Biosyst 9:2408–2416. doi:10.1039/c3mb70234k. 23884086

[28] Jiang D, Niwa M, Koong AC. 2015. Targeting the IRE1α–XBP1 branch of the unfolded protein response in human diseases. Semin Cancer Biol 33:48–56. doi:10.1016/j.semcancer.2015.04.010. 25986851 PMC4523453

[29] Madkour LH. 2020. Nanoparticles induce oxidative and endoplasmic reticulum stresses, p 329–401. Springer, Cham, Switzerland.

[30] Cross BCS, Bond PJ, Sadowski PG, Jha BK, Zak J, Goodman JM, Silverman RH, Neubert TA, Baxendale IR, Ron D, Harding HP. 2012. The molecular basis for selective inhibition of unconventional mRNA splicing by an IRE1-binding small molecule. Proc Natl Acad Sci U S A 109:E869–E878. doi:10.1073/pnas.1115623109. 22315414 PMC3326519

[31] Cheon SA, Jung K-W, Bahn Y-S, Kang HA. 2014. The unfolded protein response (UPR) pathway in *Cryptococcus*. Virulence 5:341–350. doi:10.4161/viru.26774. 24504058 PMC3956512

[32] Back SH, Schröder M, Lee K, Zhang K, Kaufman RJ. 2005. ER stress signaling by regulated splicing: IRE1/HAC1/XBP1. Methods 35:395–416. doi:10.1016/j.ymeth.2005.03.001. 15804613

[33] Weichert M, Guirao-Abad J, Aimanianda V, Krishnan K, Grisham C, Snyder P, Sheehan A, Abbu RR, Liu H, Filler SG, Gruenstein EI, Latgé J-P, Askew DS. 2020. Functional coupling between the unfolded protein response and endoplasmic reticulum/Golgi Ca^2+^-ATPases promotes stress tolerance, cell wall biosynthesis, and virulence of *Aspergillus fumigatus*. mBio 11:e01060-20. doi:10.1128/mBio.01060-20. 32487759 PMC7267887

[34] Mulder HJ, Nikolaev I, Madrid SM. 2006. HACA, the transcriptional activator of the unfolded protein response (UPR) in *Aspergillus niger*, binds to partly palindromic UPR elements of the consensus sequence 5′-CAN(G/A)NTGT/GCCT-3′. Fungal Genet Biol 43:560–572. doi:10.1016/j.fgb.2006.02.005. 16709461

[35] Mulder HJ, Saloheimo M, Penttilä M, Madrid SM. 2004. The transcription factor HACA mediates the unfolded protein response in *Aspergillus niger*, and up-regulates its own transcription. Mol Genet Genomics 271:130–140. doi:10.1007/s00438-003-0965-5. 14730445

[36] Tan S-X, Teo M, Lam YT, Dawes IW, Perrone GG. 2009. Cu, Zn superoxide dismutase and NADP(H) homeostasis are required for tolerance of endoplasmic reticulum stress in *Saccharomyces cerevisiae*. Mol Biol Cell 20:1493–1508. doi:10.1091/mbc.e08-07-0697. 19129474 PMC2649269

[37] Fjelstrup S, Andersen M, Thomsen J, Wang J, Stougaard M, Pedersen F, Ho Y-P, Hede M, Knudsen B. 2017. The effects of dithiothreitol on DNA. Sensors 17:1201. doi:10.3390/s17061201. PMC5492665 28538659

[38] Alliegro MC. 2000. Effects of dithiothreitol on protein activity unrelated to thiol–disulfide exchange: for consideration in the analysis of protein function with Cleland’s reagent. Anal Biochem 282:102–106. doi:10.1006/abio.2000.4557. 10860505

[39] Pollard MG, Travers KJ, Weissman JS. 1998. Ero1p: a novel and ubiquitous protein with an essential role in oxidative protein folding in the endoplasmic reticulum. Mol Cell 1:171–182. doi:10.1016/s1097-2765(00)80018-0. 9659914

[40] Montenegro-Montero A, Goity A, Larrondo LF. 2015. The bZIP transcription factor HAC-1 is involved in the unfolded protein response and is necessary for growth on cellulose in *Neurospora crassa*. PLoS One 10:e0131415. doi:10.1371/journal.pone.0131415. 26132395 PMC4488935

[41] Tanaka M, Shintani T, Gomi K. 2015. Unfolded protein response is required for *Aspergillus oryzae* growth under conditions inducing secretory hydrolytic enzyme production. Fungal Genet Biol 85:1–6. doi:10.1016/j.fgb.2015.10.003. 26496881

[42] Chaillot J, Tebbji F, Remmal A, Boone C, Brown GW, Bellaoui M, Sellam A. 2015. The monoterpene carvacrol generates endoplasmic reticulum stress in the pathogenic fungus *Candida albicans*. Antimicrob Agents Chemother 59:4584–4592. doi:10.1128/AAC.00551-15. 26014932 PMC4505245

[43] Gessner PK. 1995. Isobolographic analysis of interactions: an update on applications and utility. Toxicology 105:161–179. doi:10.1016/0300-483X(95)03210-7. 8571354

[44] Moreno-Martinez E, Vallieres C, Holland SL, Avery SV. 2015. Novel, synergistic antifungal combinations that target translation fidelity. Sci Rep 5:16700–16710. doi:10.1038/srep16700. 26573415 PMC4648087

[45] Carter AP, Clemons WM, Brodersen DE, Morgan-Warren RJ, Wimberly BT, Ramakrishnan V. 2000. Functional insights from the structure of the 30S ribosomal subunit and its interactions with antibiotics. Nature 407:340–348. doi:10.1038/35030019. 11014183

[46] Dean N. 1995. Yeast glycosylation mutants are sensitive to aminoglycosides. Proc Natl Acad Sci U S A 92:1287–1291. doi:10.1073/pnas.92.5.1287. 7877969 PMC42504

[47] Li Y, Zhang L, Wang D, Zhou H, Ouyang H, Ming J, Jin C. 2008. Deletion of the *msdS*/Af*msdC* gene induces abnormal polarity and septation in *Aspergillus fumigatus*. Microbiology (Reading) 154:1960–1972. doi:10.1099/mic.0.2008/017525-0. 18599824

[48] Mimura N, Fulciniti M, Gorgun G, Tai YT, Cirstea D, Santo L, Hu Y, Fabre C, Minami J, Ohguchi H, Kiziltepe T, Ikeda H, Kawano Y, French M, Blumenthal M, Tam V, Kertesz NL, Malyankar UM, Hokenson M, Pham T, Zeng Q, Patterson JB, Richardson PG, Munshi NC, Anderson KC. 2012. Blockade of XBP1 splicing by inhibition of IRE1α is a promising therapeutic option in multiple myeloma. Blood 119:5772–5781. doi:10.1182/blood-2011-07-366633. 22538852 PMC3382937

[49] Chalmers F, Mogre S, Son J, Blazanin N, Glick AB. 2019. The multiple roles of the unfolded protein response regulator IRE1α in cancer. Mol Carcinog 58:1623–1630. doi:10.1002/mc.23031. 31041814 PMC6692187

[50] Ri M, Tashiro E, Oikawa D, Shinjo S, Tokuda M, Yokouchi Y, Narita T, Masaki A, Ito A, Ding J, Kusumoto S, Ishida T, Komatsu H, Shiotsu Y, Ueda R, Iwawaki T, Imoto M, Iida S. 2012. Identification of Toyocamycin, an agent cytotoxic for multiple myeloma cells, as a potent inhibitor of ER stress-induced XBP1 mRNA splicing. Blood Cancer J 2:e79. doi:10.1038/bcj.2012.26. 22852048 PMC3408640

[51] Guillemette T, van Peij NN, Goosen T, Lanthaler K, Robson GD, van den Hondel CA, Stam H, Archer DB. 2007. Genomic analysis of the secretion stress response in the enzyme-producing cell factory *Aspergillus niger*. BMC Genomics 8:158. doi:10.1186/1471-2164-8-158. 17561995 PMC1894978

[52] Heimel K. 2015. Unfolded protein response in filamentous fungi—implications in biotechnology. Appl Microbiol Biotechnol 99:121–132. doi:10.1007/s00253-014-6192-7. 25384707

[53] Scalas D, Mandras N, Roana J, Tardugno R, Cuffini AM, Ghisetti V, Benvenuti S, Tullio V. 2018. Use of *Pinus sylvestris* L. (*Pinaceae*), *Origanum vulgare* L. (*Lamiaceae*), and *Thymus vulgaris* L. (*Lamiaceae*) essential oils and their main components to enhance itraconazole activity against azole susceptible/not-susceptible *Cryptococcus neoformans* strains. BMC Complement Altern Med 18:143. doi:10.1186/s12906-018-2219-4. 29724221 PMC5934896

[54] Rao A, Zhang Y, Muend S, Rao R. 2010. Mechanism of antifungal activity of terpenoid phenols resembles calcium stress and inhibition of the TOR pathway. Antimicrob Agents Chemother 54:5062–5069. doi:10.1128/AAC.01050-10. 20921304 PMC2981246

[55] Kim J, Chan K, Mahoney N. 2015. Augmenting the activity of monoterpenoid phenols against fungal pathogens using 2-hydroxy-4-methoxybenzaldehyde that target cell wall integrity. Int J Mol Sci 16:26850–26870. doi:10.3390/ijms161125988. 26569223 PMC4661847

[56] Niu C, Wang C, Yang Y, Chen R, Zhang J, Chen H, Zhuge Y, Li J, Cheng J, Xu K, Chu M, Ren C, Zhang C, Jia C. 2020. Carvacrol induces *Candida albicans* apoptosis associated with Ca2+/Calcineurin pathway. Front Cell Infect Microbiol 10:192. doi:10.3389/fcimb.2020.00192. 32426298 PMC7203418

[57] Tartor YH, Hassan FAM. 2017. Assessment of carvacrol for control of avian aspergillosis in intratracheally challenged chickens in comparison to voriconazole with a reference on economic impact. J Appl Microbiol 123:1088–1099. doi:10.1111/jam.13557. 28795522

[58] Nekrutenko A, He J. 2006. Functionality of unspliced *XBP1* is required to explain evolution of overlapping reading frames. Trends Genet 22:645–648. doi:10.1016/j.tig.2006.09.012. 17034899

[59] Plumb R, Zhang Z-R, Appathurai S, Mariappan M. 2015. A functional link between the co-translational protein translocation pathway and the UPR. Elife 4:2–27. doi:10.7554/eLife.07426. PMC4456659 25993558

[60] Kanda S, Yanagitani K, Yokota Y, Esaki Y, Kohno K. 2016. Autonomous translational pausing is required for *XBP1u* mRNA recruitment to the ER via the SRP pathway. Proc Natl Acad Sci U S A 113:E5886–E5895. doi:10.1073/pnas.1604435113. 27651490 PMC5056097

[61] Aragón T, van Anken E, Pincus D, Serafimova IM, Korennykh AV, Rubio CA, Walter P. 2009. Messenger RNA targeting to endoplasmic reticulum stress signalling sites. Nature 457:736–740. doi:10.1038/nature07641. 19079237 PMC2768538

[62] Yanagitani K, Kimata Y, Kadokura H, Kohno K. 2011. Translational pausing ensures membrane targeting and cytoplasmic splicing of *XBP1u* mRNA. Science 331:586–589. doi:10.1126/science.1197142. 21233347

[63] Martin D, Li Y, Yang J, Wang G, Margariti A, Jiang Z, Yu H, Zampetaki A, Hu Y, Xu Q, Zeng L. 2014. Unspliced X-box-binding protein 1 (XBP1) protects endothelial cells from oxidative stress through interaction with histone deacetylase 3. J Biol Chem 289:30625–30634. doi:10.1074/jbc.M114.571984. 25190803 PMC4215241

[64] Zhao G, Fu Y, Cai Z, Yu F, Gong Z, Dai R, Hu Y, Zeng L, Xu Q, Kong W. 2017. Unspliced XBP1 confers VSMC homeostasis and prevents aortic aneurysm formation via FoxO4 interaction. Circ Res 121:1331–1345. doi:10.1161/CIRCRESAHA.117.311450. 29089350

[65] Zhou B, Xie J, Liu X, Wang B, Pan L. 2016. Functional and transcriptomic analysis of the key unfolded protein response transcription factor HacA in *Aspergillus oryzae*. Gene 593:143–153. doi:10.1016/j.gene.2016.08.018. 27520586

[66] Thibault G, Ismail N, Ng DTW. 2011. The unfolded protein response supports cellular robustness as a broad-spectrum compensatory pathway. Proc Natl Acad Sci U S A 108:20597–20602. doi:10.1073/pnas.1117184109. 22143797 PMC3251055

[67] Fun XH, Thibault G. 2020. Lipid bilayer stress and proteotoxic stress-induced unfolded protein response deploy divergent transcriptional and non-transcriptional programmes. Biochim Biophys Acta Mol Cell Biol Lipids 1865:158449. doi:10.1016/j.bbalip.2019.04.009. 31028913

