# Supplementary figures and images for: A Human IRE1 Inhibitor Blocks the Unfolded Protein Response in the Pathogenic Fungus Aspergillus fumigatus and Suggests Noncanonical Functions within the Pathway
Source: mSphere. 2020 Oct 21;5(5):e00879-20. doi: 10.1128/mSphere.00879-20 (PMC7580959; doi:10.1128/mSphere.00879-20)

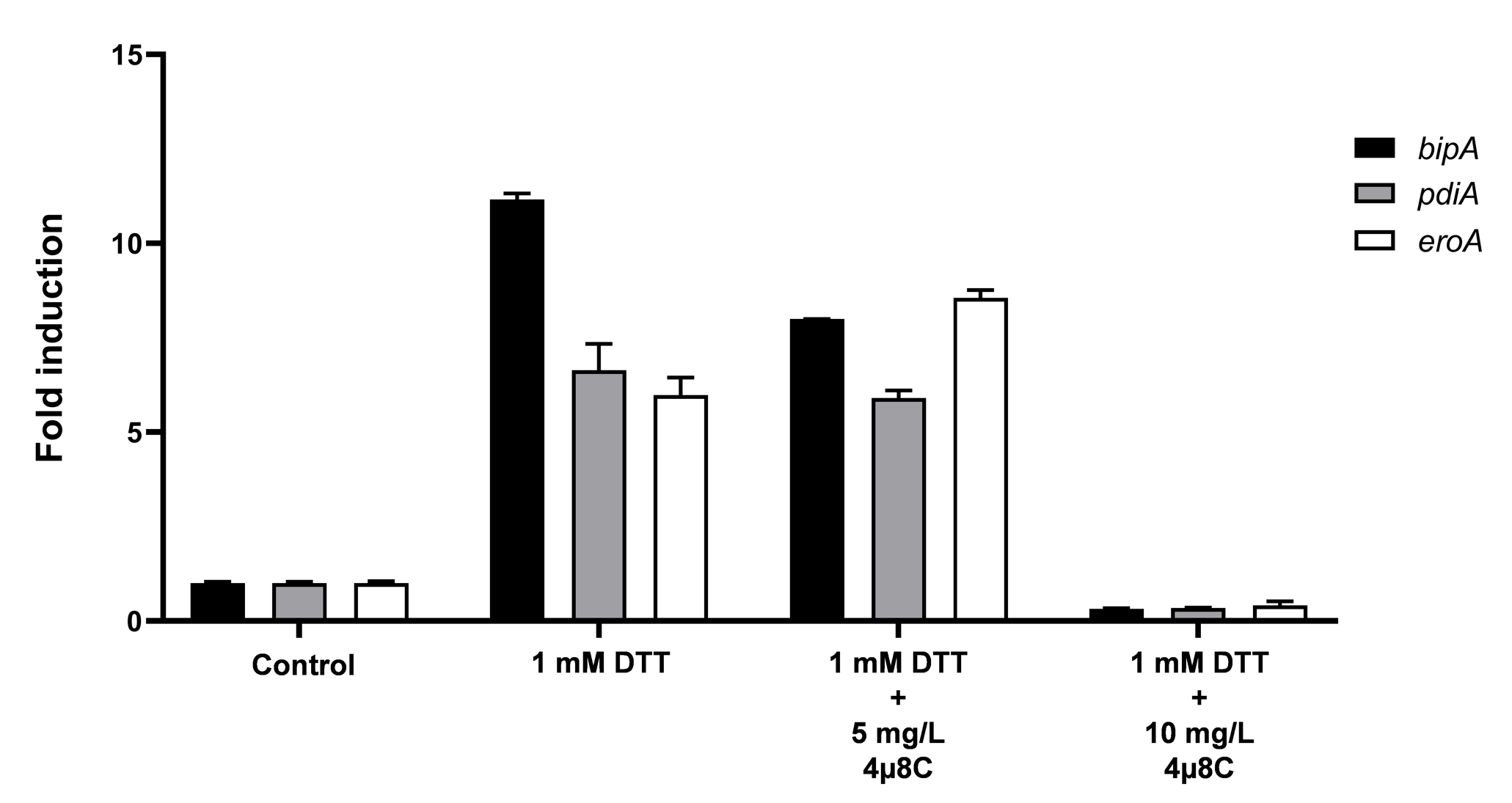

Supplement: FIG S1 [file mSphere.00879-20-sf001.tif]

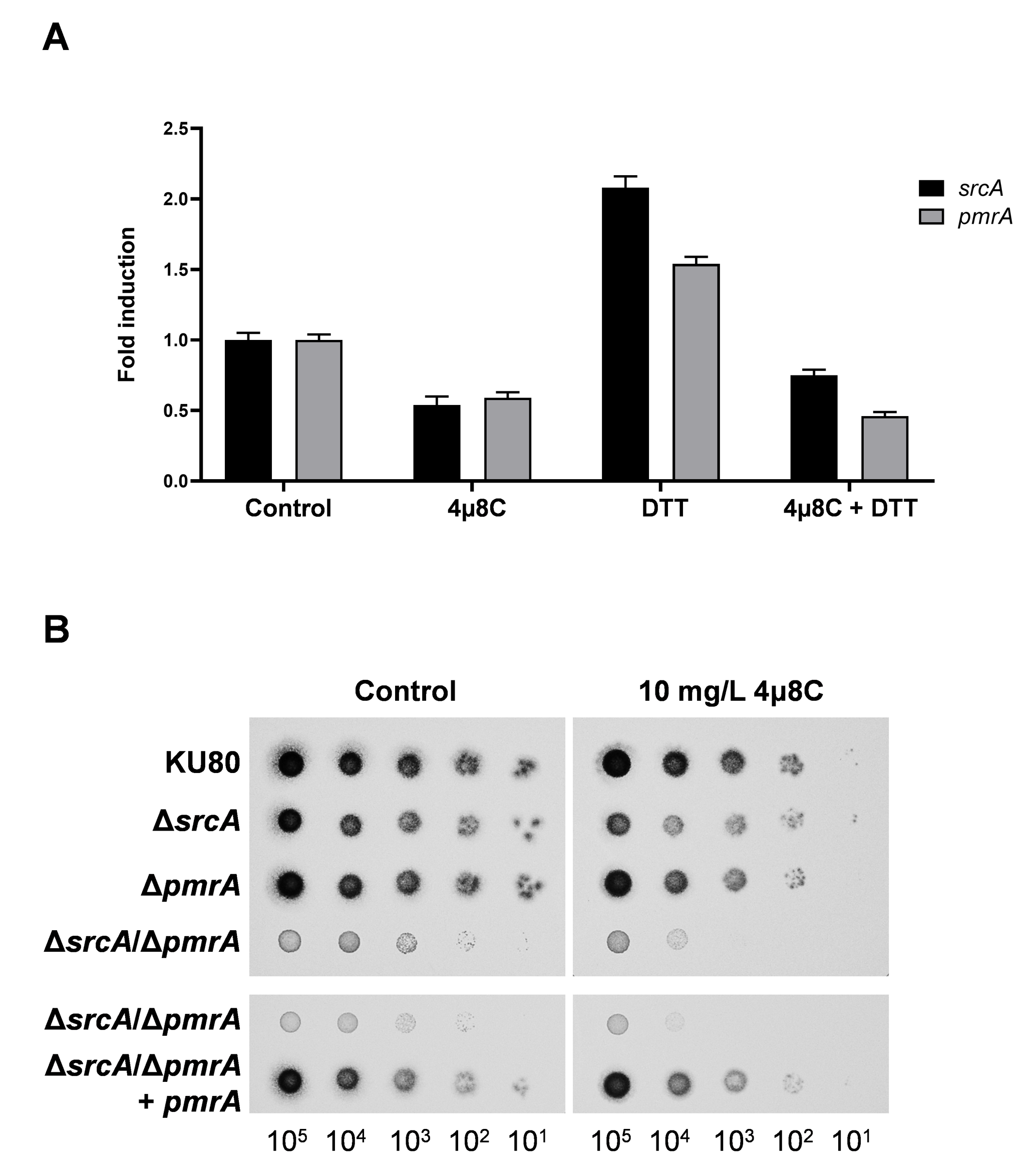

Supplement: FIG S2 [file mSphere.00879-20-sf002.tif]

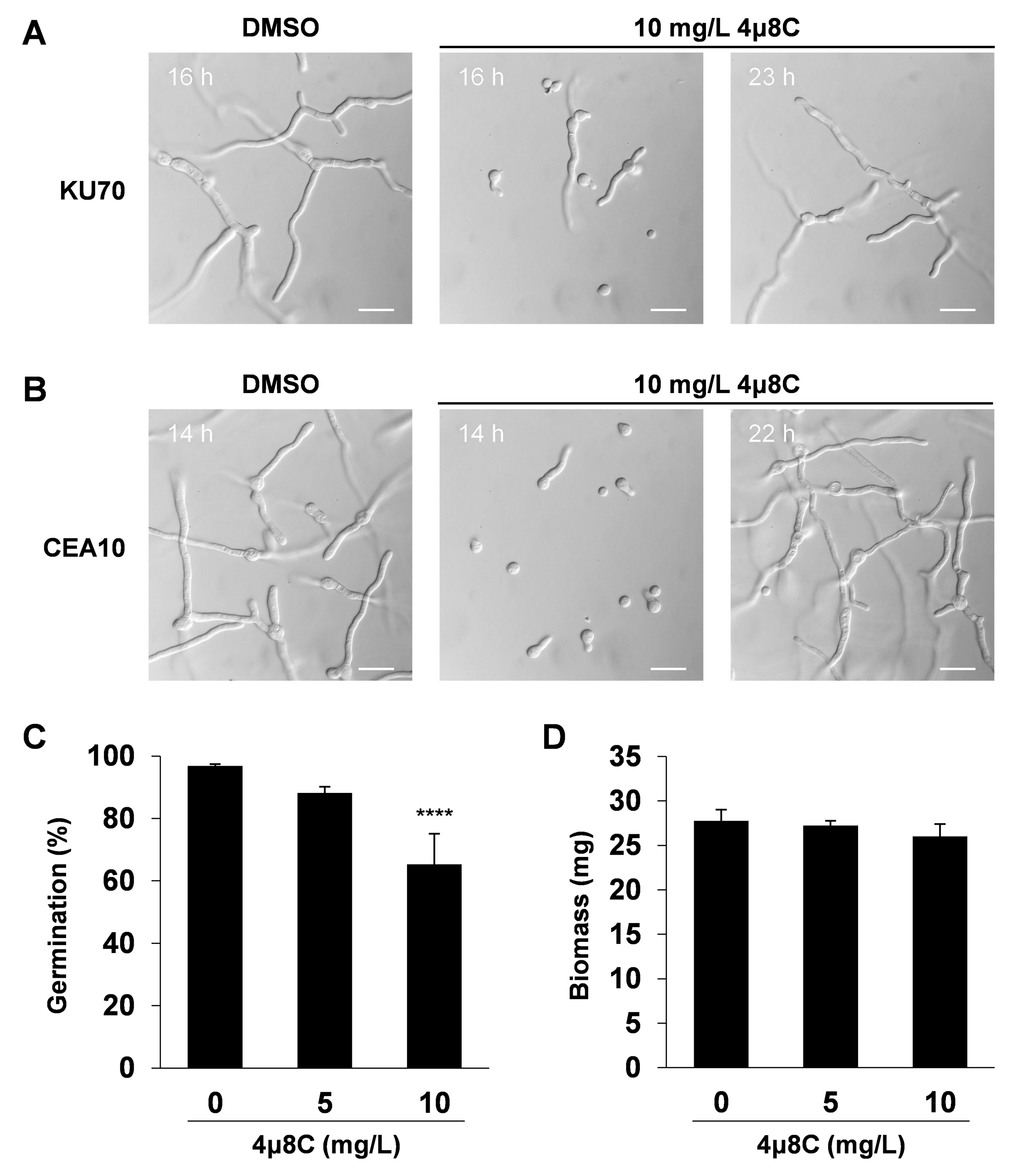

Supplement: FIG S3 [file mSphere.00879-20-sf003.tif]

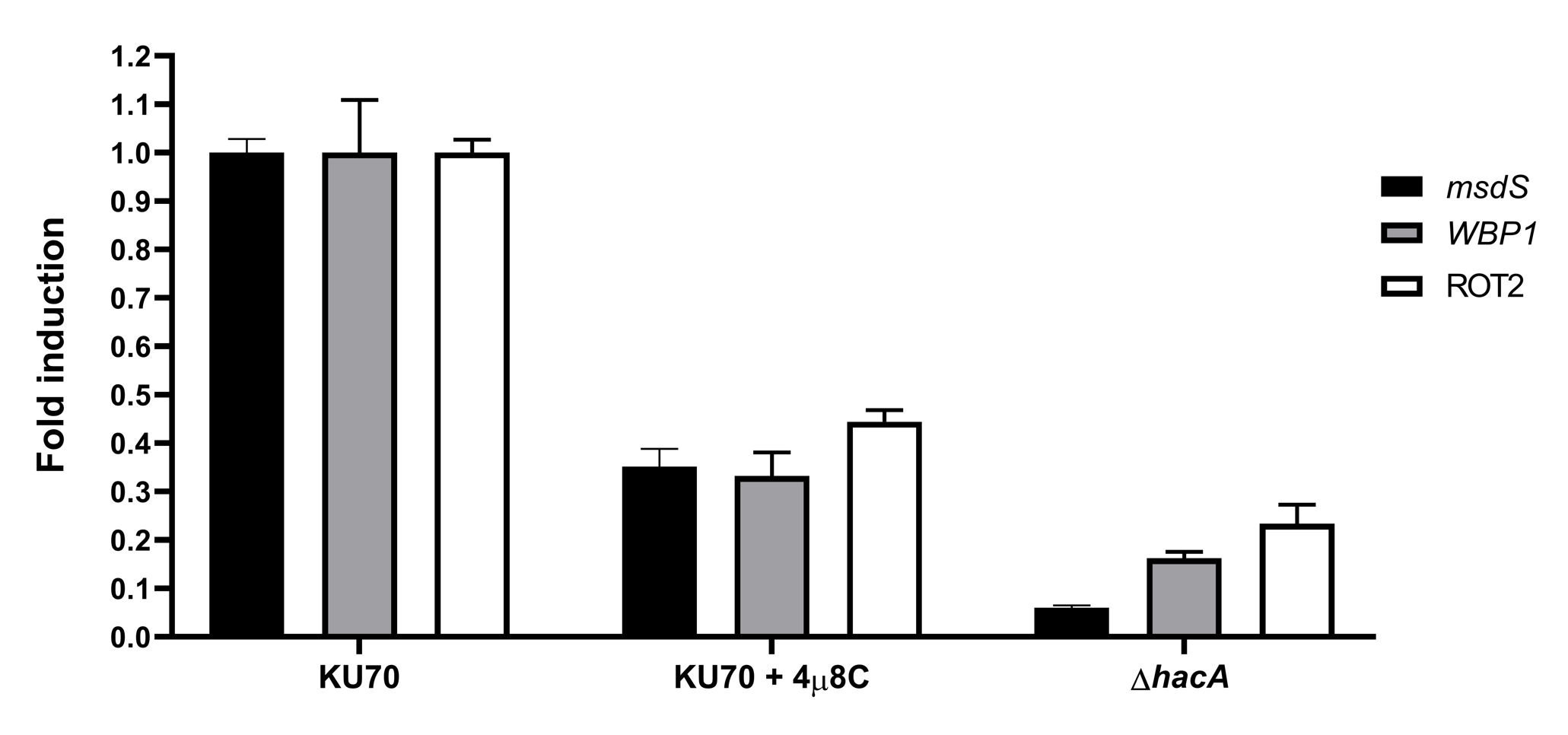

Supplement: FIG S4 [file mSphere.00879-20-sf004.tif]

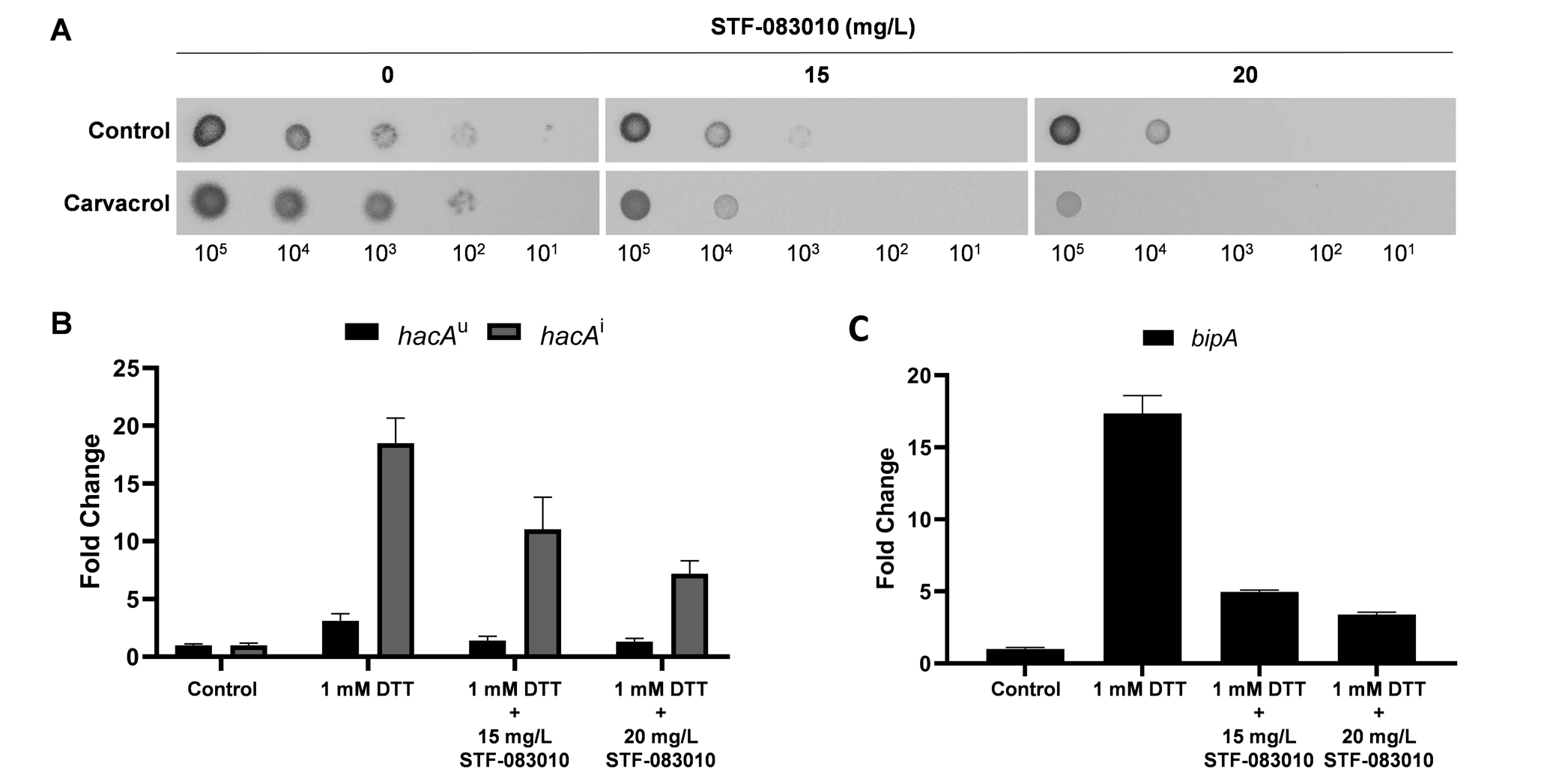

Supplement: FIG S5 [file mSphere.00879-20-sf005.tif]

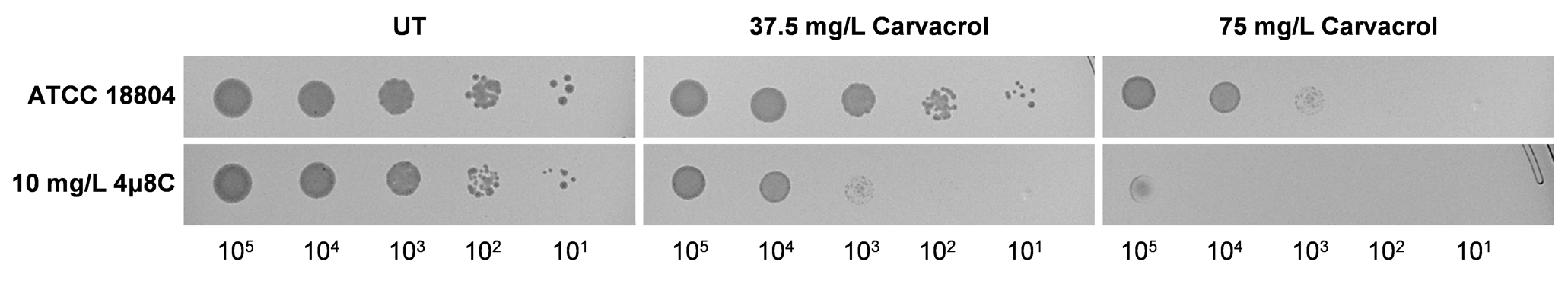

Supplement: FIG S6 [file mSphere.00879-20-sf006.tif]
