# Supplementary material for: A Human IRE1 Inhibitor Blocks the Unfolded Protein Response in the Pathogenic Fungus Aspergillus fumigatus and Suggests Noncanonical Functions within the Pathway
Source: mSphere. 2020 Oct 21;5(5):e00879-20. doi: 10.1128/mSphere.00879-20 (PMC7580959; doi:10.1128/mSphere.00879-20)
Supplement: TABLE S1 [file mSphere.00879-20-st001.docx]

| Strain | Number | Genotype or features | Origin or reference |
| --- | --- | --- | --- |
| CEA10 | 250 | Clinical isolate | Giraldin *et al*., 1993*^a^* |
| KU70 | 124 | Δ*akuA*::*ptrA* | Krappmann *et al*., 2006*^b^* |
| KU80 | 399 | ∆*akuB*::*pyrG^+^* | Da Silva *et al*., 2006*^c^* |
| Δ*hacA* | 144 | Δ*akuA*::*ptrA*, Δ*hacA*::*hph* | Reference 3 |
| Δ*hacA* | 467 | Δ*akuA*::*ptrA*, Δ*hacA*::*six* | Reference 33 |
| *ireA*∆*^RNase^* | 241 | Δ*akuA*::*ptrA*, Δ*ireA*::*ireA*^Δ10^ | Reference 4 |
| IreA* | 212 | Δ*akuA*::*ptrA*, Δ*ireA*::*ireA* | Reference 4 |
| Δ*srcA* | 402 | ∆*akuB*::*pyrG^+^*_,_ ∆*srcA*::*six* | Reference 33 |
| Δ*pmrA* | 630 | ∆*akuB*::*pyrG^+^*_,_ ∆*pmrA*::*six* | Reference 33 |
| Δ*srcA*/Δ*pmrA* | 643 | ∆*akuB*::*pyrG^+^*_,_ ∆*srcA*::*six,* ∆*pmrA*::*six-cme^R^-*β*-rec-six* | Reference 33 |
| Δ*srcA*/Δ*pmrA* + *pmrA* | 769 | ∆*akuB*::*pyrG^+^*_,_ ∆*srcA*::*six,* ∆*pmrA*::*six*-*cme^R^-*β*-rec-six, pmrA/hph* | Reference 33 |

**Table S1.** Strains of *A. fumigatus* used in this study.

*^a^*Girardin H, Latge JP, Srikantha T, Morrow B, Soll DR. 1993. J Clin Microbiol 31:1547–1554.

*^b^*Krappmann S, Sasse C, Braus GH. 2006. Eukaryot Cell 5:212–215.

*^c^*Da Silva Ferreira ME, Kress M, Savoldi M, Goldman MHS, Härtl A, Heinekamp T, Brakhage AA, Goldman GH. 2006. Eukaryot Cell 5:207–211.
