# Supplementary material for: A Human IRE1 Inhibitor Blocks the Unfolded Protein Response in the Pathogenic Fungus Aspergillus fumigatus and Suggests Noncanonical Functions within the Pathway
Source: mSphere. 2020 Oct 21;5(5):e00879-20. doi: 10.1128/mSphere.00879-20 (PMC7580959; doi:10.1128/mSphere.00879-20)
Supplement: TABLE S2 [file mSphere.00879-20-st002.docx]

**Table S2.** Oligonucleotides (primers and probes) used for gene expression analyses.

| Number | Primer name | Sequence (5’-3’) |
| --- | --- | --- |
| ***SYBR^®^ Green-based detection*** | | |
| 1252 | *18S rRNA* (F) | ACTGATACGGGGCTCTTTTG |
| 1253 | *18S rRNA* (R) | GACTTGCCCTCCAATTGTTC |
| 1218 | *bipA* (F) | TGATGAAGAGCGTCTGGTTG |
| 1219 | *bipA* (R) | TCTGGACATCCTTGTCATCG |
| 1279 | *eroA* (F) | GAGTTCCGCAATCGCTTTAG |
| 1280 | *eroA* (R) | TTCAGAGCCGTTCCATATCC |
| 1271 | *pdiA* (F) | TCAAGGTCGATTGCACTGAG |
| 1272 | *pdiA* (R) | GGAGGCAAAGTAACCGATGA |
| 1228 | *sod1* (F) | CGTCTCTTGGAACATCAAGG |
| 1229 | *sod1* (R) | TCTTGCCATAGGGGTTGAAG |
| 1442 | Afu8g05720 (F) | TGAGACGATGGCTCTAAAGG |
| 1443 | Afu8g05720 (R) | GATTACCCGCCATGTCAAAG |
| 1418 | *WBP1* (F) | TTGAGGATGTTGCAGCTACG |
| 1419 | *WBP1* (R) | TGACAGTCGCAGAAAACCAC |
| 1420 | *ROT2* (F) | GCGACGTGCATAATGTCAAC |
| 1421 | *ROT2* (R) | ATGATCGGGTCAAGACGAAG |
| 1422 | *msdS* (F) | TCAGCATCCAAACAGGTCAG |
| 1423 | *msdS* (R) | AACCGCTTGGGATCATACAC |
| 1232 | *srcA* (F) | CGGTGCTGCTACTGTTTTTG |
| 1233 | *srcA* (R) | ACTTGTGGAAATGGGACAGC |
| 1212 | *pmrA* (F) | AACCATTCGAGGTCCACAAC |
| 1213 | *pmrA* (R) | GCCGGAGAATTTGCACTAAC |
| 1355 | *hacA^u/I^* (F) | TTGTTCAAGCAAGAAGGTGATG |
| 1356 | *hacA^u^* (R) | GTCGCACAACACCGCTG |
| 1354 | *hacA^I^* (R) | ACTGACACTGCAGGATGTTGTG |
| ***TaqMan-based detection*** | | |
| - | *18S rRNA* probe | TGAGAAACGGCTACCACATCCAAGGA |
| 1257 | *18S rRNA* (F) | AACGGGTAACGGGGAATTAG |
| 1258 | *18S rRNA* (R) | GTCGGGATTGGGTAATTTGC |
| - | *hacA^u/i^* probe | CCATTTCCCACTCCCTCAATCACTGA |
| 1355 | *hacA^u/I^* (F) | TTGTTCAAGCAAGAAGGTGATG |
| 1356 | *hacA^u^* (R) | GTCGCACAACACCGCTG |
| 1354 | *hacA^I^* (R) | ACTGACACTGCAGGATGTTGTG |
